# Bilateral, Simultaneous, Acute Angle Closure Glaucoma in Pseudophakia Induced by Chlorthalidone

**DOI:** 10.1155/2016/3713818

**Published:** 2016-05-05

**Authors:** Indra Durai, Mrunali Mohan Dhavalikar, Chandran Prem Anand, Venkatraman Ganesh, Ramaswami Krishnadas

**Affiliations:** ^1^Aravind Eye Care System, Madurai, India; ^2^Aravind Eye Care System, Coimbatore, India

## Abstract

*Purpose*. To report two persons with acute, bilateral, and simultaneous angle closure glaucoma in pseudophakia secondary to uveal effusions induced by administration of chlorthalidone.* Methods*. Case reports.* Results*. Bilateral shallow anterior chambers and high intraocular pressure with decline in visual acuity were reported in two patients within days of intake of chlorthalidone for systemic hypertension. Gonioscopy confirmed appositional angle closure while choroidal detachment and ciliochoroidal detachment were revealed on ultrasonographic studies. Discontinuing chlorthalidone and institution of aqueous suppressants to reduce IOP and cycloplegics reversed angle closure and glaucoma.* Conclusions*. Reports of angle closure glaucoma in pseudophakic eyes induced by idiosyncratic reaction to chlorthalidone confirms that osmotic changes in the crystalline lens has no role in the pathogenesis of drug induced glaucoma and reaffirms that glaucoma is secondary to ciliochoroidal detachment and ciliary body rotation and edema.

## 1. Introduction

Pseudophakic angle closure glaucoma is a rare occurrence. It has been reported to frequently occur as a consequence of pupil block with iris bombe from various causes, aqueous misdirection, choroidal detachment and effusions, suprachoroidal hemorrhage, and capsular bag distension syndrome with anterior shift of the iris-lens diaphragm. Bilateral, simultaneous, acute angle closure glaucoma in pseudophakic eyes is a still rarer entity and has been reported as an idiosyncratic reaction following administration of sulphonamide containing drugs, acetazolamide and hydrochlorothiazide [1, 2]. We report a series of two patients, both pseudophakic in either eye, who had simultaneous acute angle closure glaucoma following intake of chlorthalidone for management of systemic hypertension.

## 2. Case Description

### 2.1. Report of Case 1

A 71-year-old male presented with complaints of sudden blurring of vision in both eyes associated with pain and coloured haloes since morning. He had had bilateral, uneventful clear corneal phacoemulsification with posterior chamber intraocular lens implantation five years earlier with no other antecedent ocular disease. Medical history was significant for systemic hypertension for which he was undergoing treatment for the past 15 years. He was prescribed Klorzid tablets (chlorthalidone 12.5 mg, German Remedies Limited, Zydus Cadila) for systemic hypertension and AB Phylline tablets for bronchitis for the week preceding his eye consultation.

**Figure 1:**
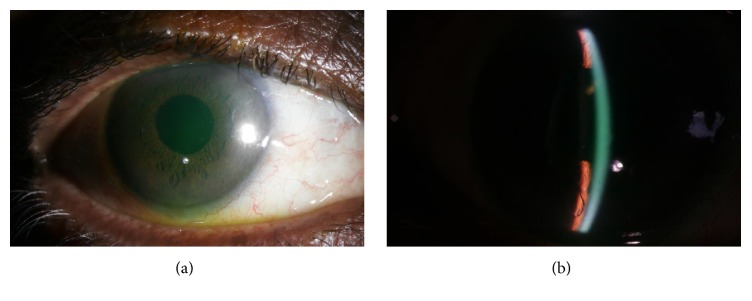
Slit lamp photograph of the left eye of the patient in Case Report 1 with shallow anterior chambers.

**Figure 2:**
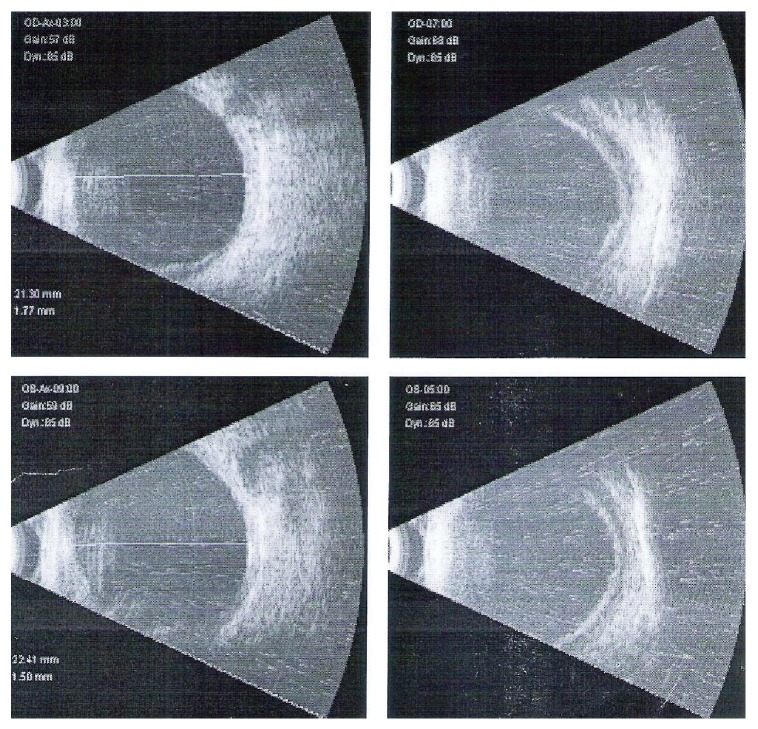
B-scan USG of patient in Case Report 1 with choroidal detachments on presentation.

Examination revealed uncorrected acuity of 6/60 (20/200) OU. Vision was correctable to 20/20 OU with −3.75 D Sphere OD and −2.50 D Sphere OS, respectively, with near vision N6 with +2.50 addition in both eyes. Anterior segment examination revealed corneal epithelial edema, shallow anterior chambers, sluggishly reacting pupils, and posterior chamber intraocular lenses in both eyes (Figures 1(a) and 1(b)). Evaluation of fundus revealed normal optic discs and posterior pole. Intraocular pressure (IOP) was 41 mmHg OD and 36 mmHg OS, respectively, by Goldmann applanation tonometry. Gonioscopy performed with a Goldmann 2 mirror revealed 360-degree closed angles with appositional angle closure and no angle structures visible. Ultrasound biomicroscopic examination revealed shallow anterior chamber with ciliary body detachment. B-scan ultrasonography revealed 360-degree peripheral shallow choroidal detachment (Figure 2). A tentative diagnosis of chlorthalidone induced bilateral angle closure glaucoma secondary to ciliochoroidal effusions was reached and treatment initiated with brimonidine eye drops OU and cycloplegic homatropine eye drops OU was also started thrice a day. Chlorthalidone which has been reported to cause idiosyncratic angle closure glaucoma was presumed to be the cause of choroidal effusion and consequent angle closure and hence discontinued. Oral acetazolamide was avoided since it is known that ciliary-choroidal effusion could be caused or aggravated by cross-reactivity due its sulphonamide component. When the patient reported two days later, his uncorrected vision remained at 20/200 OU, but IOP was 20 and 18 mmHg in the right and left eyes. Anterior segment examination revealed clear corneas and shallow anterior chambers (OD > OS). Fundus examination was normal in both eyes. Gonioscopy revealed closed, angle configuration OU. UBM and B-scan ultrasonography were characteristic of ciliary body and choroidal detachments, as observed earlier. Treatment with topical aqueous suppressants and cycloplegics was continued. A week later, the patients uncorrected visual acuity was 20/30 OD and 20/20 OS. Vision improved to 20/20 OU with −1.00 × 90 D cylinder OD and −0.50 × 90 OS, respectively. Anterior segment examination revealed clear corneas and deep anterior chambers OU. Fundus examination was normal in both eyes. Intraocular pressure (IOP) was 8 mmHg OD and 11 mmHg OS. Gonioscopy revealed wide open angles OU. UBM and B-scan ultrasonography revealed 360-degree resolving choroidal detachments. The topical medications were then discontinued. The patient was advised of periodical evaluation to exclude any residual glaucoma.

### 2.2. Report of Case 2

**Figure 3:**
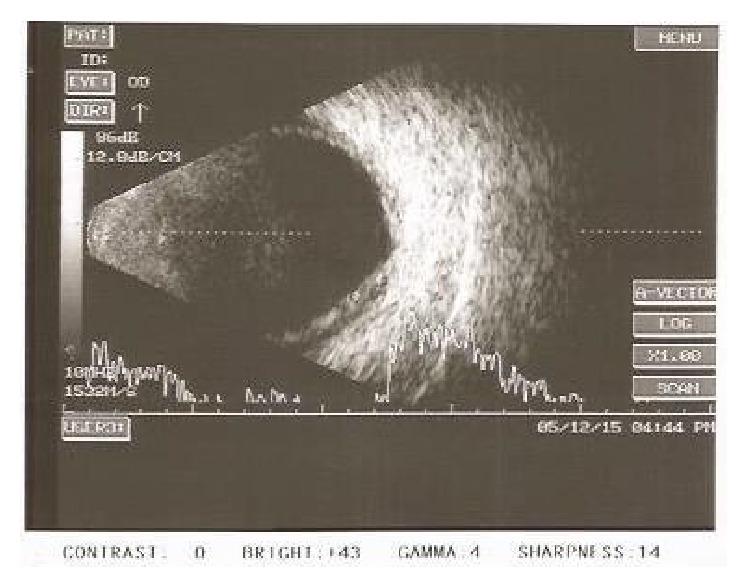
Right eye of the patient in Case Report 2: B-scan ultrasonography shows shallow choroidal detachment.

**Figure 4:**
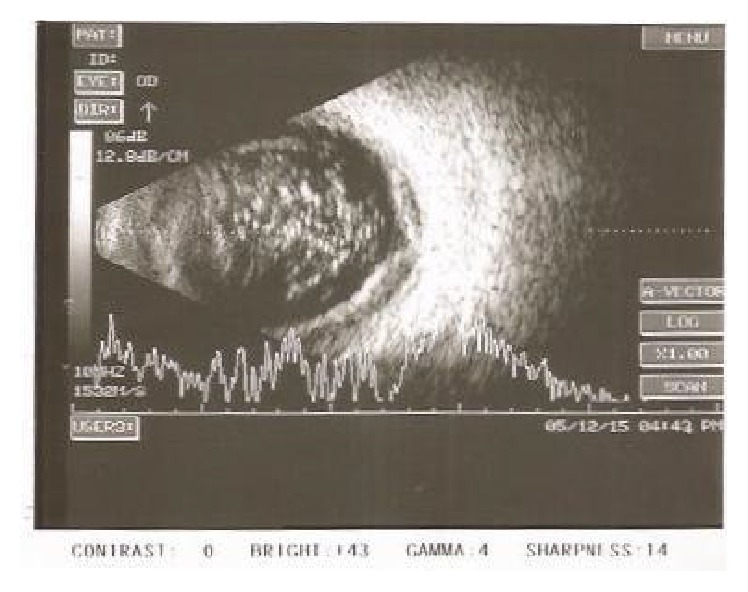
Left eye B-scan of Patient 2: ultrasonography shows asteroid hyalosis and shallow choroidal detachment.

**Figure 5:**
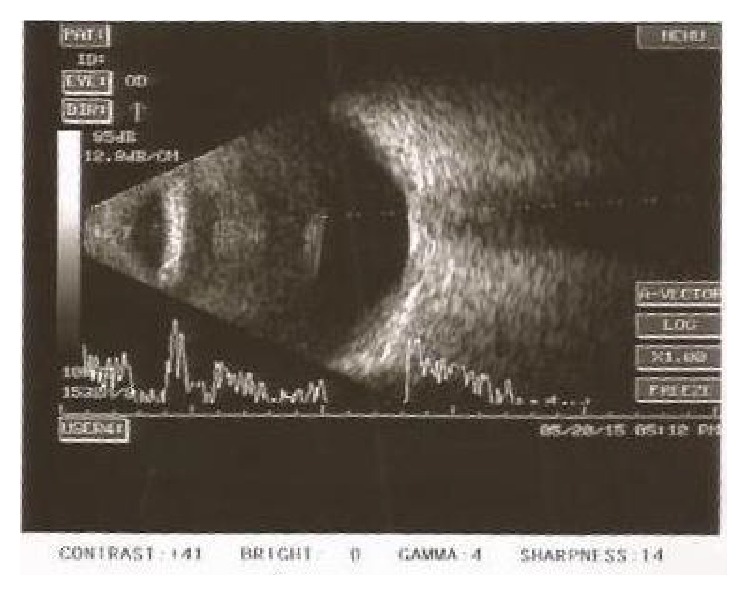
Right eye (Patient 2) B-scan ultrasosonography shows normal features suggesting resolution of choroidal detachment following withdrawal of chlorthalidone.

**Figure 6:**
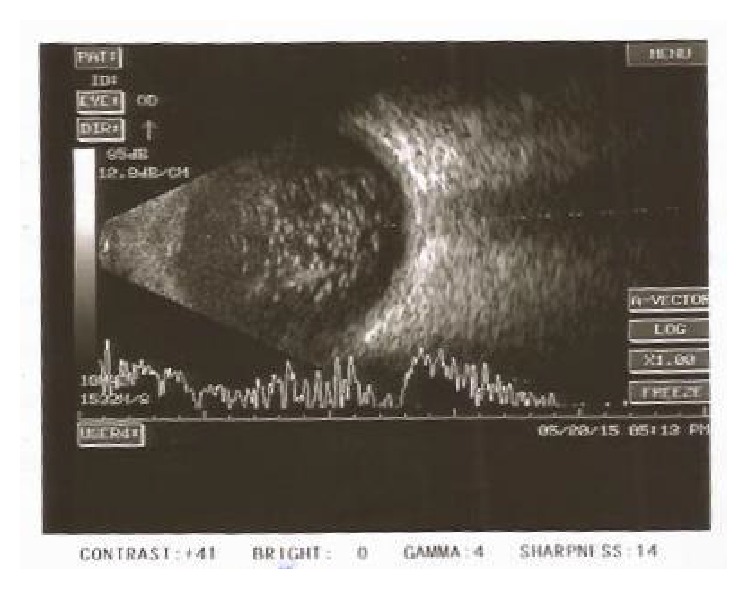
Left eye (Patient 2) B-scan ultrasonography shows resolution of choroidal detachment.

A 65-year-old male patient was referred to glaucoma services with complaints of sudden and painless diminution of vision associated with severe headache and nausea for 3 days. He had been treated with a fixed combination of timolol and brimonidine twice a day and Bimatoprost 0.01% once at night elsewhere. He was a known patient of diabetes mellitus on treatment for 30 years. He had uneventful phacoemulsification with intraocular lens implantation in both eyes 5–7 months earlier than onset of his symptoms. On examination his visual acuity was 6/12 improving with −0.75 D cylinder at 90° to 6/9 in the right eye and 6/24 improving with −1.25 D sphere and −0.50 D cylinder at 120° to 6/9 in the left eye. His anterior segment examination revealed clear corneal incision scars, anterior chamber depth was slightly shallow, iris was chaffed in the region of the corneal incision, and the pupil was round and acting in a sluggish way with posterior chamber intraocular lens in situ. His intraocular pressure was 4 mmHg and 8 mmHg in the right and left eyes, respectively. Gonioscopy revealed narrow angles in both eyes, open without synechiae on indentation. The fundus revealed normal sized optic nerve with healthy neuroretinal rim and a CD ratio of 0.5 : 1 in both eyes. Bilateral moderate nonproliferative diabetic retinopathy with asteroid hyalosis in the left eye and bilateral peripheral moderate serous choroidal detachments were observed. On further enquiry the patient revealed that his physician had added chlorthalidone tablets 25 mg once a day for the control of hypertension three days prior to the onset of symptoms. A provisional diagnosis of drug (chlorthalidone) induced and acute bilateral angle closure with choroidal detachment was reached and the drug chlorthalidone was discontinued. A week later, visual acuity had improved to 6/9 OU and his anterior chamber had deepened. His intraocular pressure was 10 mmHg and 8 mmHg in the right and left eyes, respectively. Peripheral choroidal detachments persisted but reduced considerably and were confirmed by ultrasonography B-scan (Figures 3 and 4). He was advised to discontinue Bimatoprost and report for review after a week. On his last visit about 15 days after the onset of symptoms the visual acuity was 6/9 OU. The anterior chamber had deepened considerably. His pupils were normal. The intraocular pressure was 13 mmHg OU. Gonioscopy revealed wide open angles. The trabecular meshwork was normally pigmented and there were intermittent areas of excess pigment deposition over the anterior chamber angle. The fundus appeared normal and serous choroidal detachments had resolved completely in both eyes, confirmed by ultrasonography B-scan (Figures 5 and 6). He was advised to discontinue timolol-brimonidine combination and report for review periodically to exclude recurrence/residual glaucoma.

## 3. Comments/Discussion

Transient acute myopia and angle closure glaucoma secondary to uveal effusions have been reported after administration of several medications containing sulphonamide group [3]. Chlorthalidone, a monsosulfomyl containing sulphonamide diuretic used in treatment of systemic hypertension, has been reported to cause acute myopia [4]. There has, however, been only a single report of bilateral angle closure glaucoma induced by chlorthalidone [5], to the best of the knowledge of the authors, although it has been known earlier that chlorthalidone can potentially precipitate to bilateral acute angle closure glaucoma owing to uveal effusion as a consequence of idiosyncratic drug toxicity. Transient myopia and ciliochoroidal detachment following administration of chlorthalidone have also been widely reported earlier. Sulphonamide induced secondary angle closure glaucoma is also not unknown in eyes following uneventful cataract surgery and intraocular lens implantation [1, 2]. The current series of two persons with bilateral, simultaneous angle closure glaucoma in pseudophakia has been reported with the purpose of adding to the already vast repertoire of bilateral angle closure glaucoma caused by idiosyncratic drug induced uveal effusions. Accommodative spasm and increase in lens thickness [3] have been postulated as one of the mechanisms of acute angle closure glaucoma induced by sulphonamides, but reports of acute angle closure glaucoma in pseudophakic eyes seem to suggest mechanisms independent of the crystalline lens. Earlier reports of acute myopia in those administered chlorthalidone had postulated mechanisms related to disturbance in osmotic state of the lens and concomitant alteration of the refractive index with ultrasonographic evidence of increase in lens thickness [6]. Recent reports have, however, reinforced that alterations in lens thickness accounted for only minimal amount of anterior chamber decrease which is instead, predominantly, due to ciliochoroidal effusion [7–10].

Although the sulphonamide group of drugs have been widely reported to cause acute myopia and angle closure glaucoma, there have so far been no reports of bilateral angle closure glaucoma induced by use of topical dorzolamide and brinzolamide widely employed in management of glaucoma, though both are sulphonamide containing drugs. The widely hypothesized mechanism of angle closure induced by sulphonamides involves an idiosyncratic reaction in uveal tissues to systemic administration of many classes of drugs including sulphonamide derivative antibiotics, diuretics, and antihypertensive class of medications and is associated with expansion of extracellular space of the choroid and ciliary body. It seems most likely that it is an expansion of the extravascular compartment, due perhaps to a sudden breakdown of the blood-ocular barrier to large proteins. Ultrasound studies performed in topiramate induced angle closure had reported anterior chamber shallowing in drug induced angle closure which is predominantly due to ciliochoroidal effusion [7]. As the uveal tract continuously expands, as with continued administration of the causative drug, there is forward movement of the crystalline lens or pseudophakia-iris diaphragm, which results in further shallowing of the anterior chamber, closure of the trabecular outflow pathway by appositional closure of the peripheral iris, and marked elevation of intraocular pressure from acute angle closure.

Bilateral, simultaneous, acute angle closure glaucoma in pseudophakic eyes due to either pupillary block or aqueous misdirection is extremely uncommon and seldom reported in literature. The constellation of signs and symptoms including acute myopia, shallow chamber due to anterior shift of iris-lens diaphragm, and sudden elevation in intraocular pressure due to acute angle closure should raise clinical suspicion of idiosyncrasy related reaction from ingestion of drugs known to cause ciliary body edema, ciliochoroidal effusion, and angle closure. Such clinical findings should immediately prompt a detailed systemic medication history and diagnostic testing including B-scan ultrasonography, UBM, and anterior segment optical coherence tomography to evaluate uveal effusions, ciliary body edema, and anterior shift in the iris-lens diaphragm with appositional angle closure. For example, in the second case in our series, the patient had not volunteered information on change of medication for control of hypertension and switching over to chlorthalidone by his physician was revealed only on detailed questioning on history of systemic intake of medications. A high index of suspicion and early recognition of drug induced angle closure glaucoma are crucial since discontinuation of the causative drug along with institution of cycloplegia, if necessary with topical or systemic corticosteroids to suppress inflammation, and aqueous suppressants to lower intraocular pressure promptly reverses glaucoma and angle closure. In our case series, in both patients, the angles of the anterior chamber opened up completely without evidence of synechiae in either eye, suggesting the importance of early identification and discontinuation of the offending drug. It is important to avoid administration of miotics since these drugs worsen ciliary body edema and anterior rotation of ciliary processes accentuating angle closure glaucoma. Laser iridotomy, which is cornerstone of management of pupillary block angle closure, is seldom beneficial in drug induced angle closure glaucoma. Refractory angle closure necessitating choroidal drainage [11] to reverse glaucoma is seldom seen in drug induced angle closure glaucoma with conservative therapy reversing angle closure and glaucoma in most individuals.
